# Development of Structural Covariance From Childhood to Adolescence: A Longitudinal Study in 22q11.2DS

**DOI:** 10.3389/fnins.2018.00327

**Published:** 2018-05-18

**Authors:** Corrado Sandini, Daniela Zöller, Elisa Scariati, Maria C. Padula, Maude Schneider, Marie Schaer, Dimitri Van De Ville, Stephan Eliez

**Affiliations:** ^1^Developmental Imaging and Psychopathology Laboratory, University of Geneva School of Medicine, Geneva, Switzerland; ^2^Institute of Bioengineering, École Polytechnique Fédérale de Lausanne, Lausanne, Switzerland; ^3^Department of Neuroscience, Center for Contextual Psychiatry, Research Group Psychiatry, KU Leuven, Leuven, Belgium; ^4^Department of Radiology and Medical Informatics, University of Geneva, Geneva, Switzerland; ^5^Department of Genetic Medicine and Development, University of Geneva School of Medicine, Geneva, Switzerland

**Keywords:** schizophrenia, graph theory, connectome, synaptic stabilization, cortical development, executive functions, structural covariance

## Abstract

**Background:** Schizophrenia is currently considered a neurodevelopmental disorder of connectivity. Still few studies have investigated how brain networks develop in children and adolescents who are at risk for developing psychosis. 22q11.2 Deletion Syndrome (22q11DS) offers a unique opportunity to investigate the pathogenesis of schizophrenia from a neurodevelopmental perspective. Structural covariance (SC) is a powerful approach to explore morphometric relations between brain regions that can furthermore detect biomarkers of psychosis, both in 22q11DS and in the general population.

**Methods:** Here we implement a state-of-the-art sliding-window approach to characterize maturation of SC network architecture in a large longitudinal cohort of patients with 22q11DS (110 with 221 visits) and healthy controls (117 with 211 visits). We furthermore propose a new clustering-based approach to group regions according to trajectories of structural connectivity maturation. We correlate measures of SC with development of working memory, a core executive function that is highly affected in both idiopathic psychosis and 22q11DS. Finally, in 22q11DS we explore correlations between SC dysconnectivity and severity of internalizing psychopathology.

**Results:** In HCs network architecture underwent a quadratic developmental trajectory maturing up to mid-adolescence. Late-childhood maturation was particularly evident for fronto-parietal cortices, while Default-Mode-Network-related regions showed a more protracted linear development. Working memory performance was positively correlated with network segregation and fronto-parietal connectivity. In 22q11DS, we demonstrate aberrant maturation of SC with disturbed architecture selectively emerging during adolescence and correlating more severe internalizing psychopathology. Patients also presented a lack of typical network development during late-childhood, that was particularly prominent for frontal connectivity.

**Conclusions:** Our results suggest that SC maturation may underlie critical cognitive development occurring during late-childhood in healthy controls. Aberrant trajectories of SC maturation may reflect core developmental features of 22q11DS, including disturbed cognitive maturation during childhood and predisposition to internalizing psychopathology and psychosis during adolescence.

## Introduction

Mounting evidence has suggested that schizophrenia arises from a disorder of brain development (Feinberg, 1982; Weinberger, 1987; Crow et al., 1989; Murray et al., 1991; Insel, 2010; Rapoport et al., 2012). Indeed, psychosis typically emerges when critical brain maturation is still underway, during late-adolescence and early-adulthood. Moreover, recent work has highlighted that cognitive deficits, which represent a core dimension of schizophrenia, can manifest as early as childhood (Kremen et al., 2010; Gur et al., 2014) and help predict subsequent emergence of psychosis (Riecher-Rössler et al., 2009; Seidman et al., 2016). The neurodevelopmental model carries at least two critical implications. Firstly, it predicts that, for therapeutic interventions to be truly effective, they should target neurodevelopmental events that precede clinical manifestations (Marín, 2016; Millan et al., 2016). Secondly, it implies that biomarkers of vulnerability to psychosis can potentially manifest earlier than disease onset, in form of atypical neurodevelopmental trajectories (Weinberger, 1987; Insel, 2010; Rapoport et al., 2012). An improved characterization of such neurodevelopmental biomarkers could prove critical for informing future therapeutic interventions (Millan et al., 2016).

In the field of schizophrenia, converging evidence has pointed to the role of disturbed structural and functional connectivity (Stephan et al., 2009; van den Heuvel and Fornito, 2014) in the pathogenesis of the disease. At the most basic anatomical scale, neuropathological studies have consistently reported synaptic alterations, particularly in prefrontal regions (Garey et al., 1998; Glantz and Lewis, 2000; Rosoklija et al., 2000; Black et al., 2004; Glausier and Lewis, 2013) that are thought to arise from excessive synaptic pruning during adolescence (Feinberg, 1982; Sekar et al., 2016). Neuroimaging allows to investigate brain connectivity *in-vivo* and at a higher anatomical scale. For instance, using diffusion-weighted MRI it is possible to investigate white-matter connectivity non-invasively throughout the entire brain (Beaulieu and Allen, 1994; Basser and Pierpaoli, 1996; Mori and Barker, 1999; Basser et al., 2000; Mori and van Zijl, 2002), while functional MRI and EEG allow to explore patterns of synchronized activity underlying functional communication between brain regions (Vértes and Bullmore, 2015). Connectomics analysis can then characterize non-trivial aspects of network architecture with tools from graph theory (Sporns et al., 2005; Hagmann et al., 2007; Bullmore and Sporns, 2009; Rubinov and Sporns, 2010). Such an approach has demonstrated that healthy structural and functional brain networks find a balance between the local segregation of sub-communities of densely connected regions and the overall integration of multiple sub-networks (Sporns, 2013). This optimal organization, also known as community structure (van den Heuvel et al., 2009; Park and Friston, 2013) has shown to underlie higher cognitive performance in healthy controls (HCs) (Langer et al., 2012; Hilger et al., 2017). In patients with psychosis, on the contrary, this optimal balance is altered, with insufficient architectural integration and excessive segregation (van den Heuvel and Fornito, 2014). Moreover insufficient brain network integration might at least partially account for cognitive symptoms of schizophrenia (Langer et al., 2012; Alloza et al., 2017). So far however, little is known about how network architecture matures in children and adolescents who are at risk for developing psychosis. The characterization of such developmental trajectories could prove informative, particularly in the context of the neurodevelopmental model of schizophrenia (Insel, 2010; Rapoport et al., 2012; Marín, 2016).

22q11.2 Deletion Syndrome (22q11DS) is a powerful model to investigate the pathogenesis of psychosis from a neurodevelopmental perspective (Jonas et al., 2014). Indeed, patients with 22q11DS are at a very high risk for psychosis, with up to 30% of patients developing schizophrenia by adulthood and up to 80% presenting prodromal psychotic symptoms, typically during adolescence (Schneider et al., 2014a; Tang et al., 2014). Furthermore, compared to idiopathic psychosis, patients are typically diagnosed prior to psychiatric manifestations due to a complex somatic phenotype (McDonald-McGinn et al., 2015). 22q11DS offers therefore a unique opportunity to investigate connectivity development in young patients at risk for psychosis. 22q11DS is also characterized by insufficient maturation of executive functions, starting during childhood (Maeder et al., 2016) and recapitulating deficits in executive functions observed in idiopathic psychosis (Seidman et al., 2016). Moreover cognitive decline during childhood can predict subsequent emergence of psychosis in 22q11DS (Vorstman et al., 2015). However the relationship between brain network development and cognitive dysmaturation has not yet been investigated in 22q11DS.

Structural covariance (SC) is a powerful morphometric approach to investigate brain connectivity (Alexander-Bloch et al., 2013). This technique measures how the morphology of different brain regions is correlated across populations, based on the observation that regions connected, either functionally or by white-matter tracts, also tend to co-vary in their morphology (Alexander-Bloch et al., 2013). Several mechanisms are thought to contribute to this phenomenon (Alexander-Bloch et al., 2013), including the mutually trophic effect of axonal connections (Burgoyne et al., 1993; Gong et al., 2012), coordinated activity induced plasticity (Draganski et al., 2004; Driemeyer et al., 2008; Dehaene et al., 2010) or common genetic influences (Pezawas et al., 2005; Schmitt et al., 2008, 2009, 2010). The emergence of SC networks has moreover shown to be functionally relevant, given that the optimal organization of SC network architecture was associated with higher cognitive performance in healthy and clinical pediatric populations (Bonilha et al., 2014; Khundrakpam et al., 2016). Importantly, networks reconstructed using SC are altered in patients suffering from psychosis, with insufficient architectural integration and excessive segregation (Bassett et al., 2008; Zhang et al., 2012). Similar architectural disturbances of SC networks have been recently replicated in 22q11DS, with specific alterations affecting patients with psychotic symptoms (Sandini et al., 2017). However, so far, little is known about how SC network architecture matures in children and adolescents with 22q11DS and how this relates to cognitive maturation.

Methods to investigate the development of SC have to date mostly consisted of comparisons between age bins (Zielinski et al., 2010; Khundrakpam et al., 2013; Nie et al., 2013). Indeed, SC inherently relies on a group of subjects and thus cannot be retrieved at an individual level (Alexander-Bloch et al., 2013). Age-bin comparisons have contributed significantly to understanding the development of SC in HCs, highlighting for example differential maturation across cortical regions (Zielinski et al., 2010) and significant late-childhood architectural reorganization (Khundrakpam et al., 2013). This technique however presents several methodological limitations. Firstly, age-bin definition is arbitrary, potentially leading to inconsistencies across studies (Richmond et al., 2016). Secondly, temporal resolution is generally insufficient to truly characterize developmental trajectories. In an effort to overcome these limitations recent work has implemented a sliding-window approach, originally developed to investigate dynamics of functional connectivity, to study the development of structural covariance (Váša et al., 2018). The higher temporal resolution offered by such approach demonstrated non-linear maturation of SC networks during adolescence (Váša et al., 2018). This study was however constrained by its cross-sectional nature and in that it did not cover the childhood age range. Indeed particularly late childhood was previously shown to be a critical period for the maturation of both SCNs (Khundrakpam et al., 2013) and cognitive performance (Chelune and Baer, 1986; Anderson, 2002; Crone et al., 2006). Moreover no link with behavioral development was made.

Here we employ a sliding-window technique to investigate structural covariance network (SCN) development in a large longitudinal cohort of children and adolescents with 22q11DS and HCs. We propose a novel approach to cluster regions according to developmental trajectories of structural connectivity strength. Furthermore we correlate maturation of SCNs with development of working memory (WM), a core executive function that is highly affected in idiopathic psychosis. Finally in 22q11DS we explore correlations between SC dysconnectivity and severity of internalizing symptoms. Indeed high internalizing psychopathology represents a hallmark of the psychiatric phenotype of 22q11DS (Shashi et al., 2012; Klaassen et al., 2013, 2015) and higher internalizing symptoms such as anxiety represent a risk factor for psychosis in 22q11DS (Gothelf et al., 2007, 2013).

We hypothesized that in HCs SCNs would undergo non-linear maturation with critical reorganization during late childhood and progressive fine-tuning during adolescence, recapitulating previous findings in general population (Khundrakpam et al., 2013; Váša et al., 2018). We hypothesized that network architecture and particularly fronto-parietal connectivity maturation would be associated with WM performance. In patients we expected to observe aberrant developmental trajectories of particularly frontal connectivity given previous reports of frontal dysmaturation in 22q11DS (Schaer et al., 2009) and idiopathic psychosis (Wood et al., 2008). We expected that previously reported disturbed network architecture would emerge during adolescence, since adolescence is a critical period of vulnerability to psychosis in the general population (Insel, 2010) and in 22q11DS (Schneider et al., 2014a,b). We hypothesize that SC dysconnectivity would be associated with higher internalizing psychopathology.

## Methods

### Participants

All participants with 22q11DS were recruited at the Geneva School of Medicine in the context of a prospective longitudinal study [details about recruitment can be found in (Schaer et al., 2009; Maeder et al., 2016)].

For the present work our cohort consisted of 110 patients with 22q11DS, followed up for an average of 2 time-points (varying from 1 to 4) amounting to 221 visits. Prior to selecting our cohort structural brain scans were visually inspected to screen for the presence of gross morphological abnormalities, leading to the exclusion of two patients with 22q11DS presenting with polymicrogyria. Additionally, we recruited 117 (M/F = 58/59) HCs that were followed for an average of 1.8 time-points (varying from 1 to 4), amounting to 211 visits. Groups did not differ in terms of mean age (*p* = 0.1), time between visits, (*p* = 0.35), gender (*p* = 0.63) or handedness (*p* = 0.61). Only full-scale IQ was significantly lower in patients compared to controls (*p* < 0.001). For demographic details see Supplementary Table 1 and Supplementary Figure 1. Prevalence of main psychiatric and neurological diagnoses are reported in Supplementary Table 2. Written informed consent was obtained for all participants, and the study was approved by the Institutional Review Board of the Geneva University School of Medicine.

### Image acquisition and processing

T1-weighted images were acquired with a three-dimensional volumetric pulse sequence with a Philips 1.5T Intera scanner (sequence parameters: TR = 35 ms, TE = 6 ms, flip angle = 45°, NEX = 1, matrix size = 256 × 192, field of view = 24 cm^2^, slice thickness = 1.5 mm, 124 slices) and Siemens Trio or Prisma 3T scanners (sequence parameters for 3T scanners: TR = 2,500 ms, TE = 3 ms, flip angle = 8°, acquisition matrix = 256 × 256, field of view = 22 cm, slice thickness = 1.1 mm, and 192 slices). A test-retest approach has previously demonstrated high consistency for morphological measures across scanners (Mutlu et al., 2013). Furthermore scanner type did not differ significantly across populations (*p* = 0.14 see demographic table) and any potential effects of scanner were rigorously accounted for during statistical analysis.

Images were imported in FreeSurfer software package (http://surfer.nmr.mgh.harvard.edu/fswiki) for a precise and semi-automatic reconstruction of the internal and external cortical surfaces (Fischl and Dale, 2000). The mean cortical thickness of 148 brain regions was then computed for each scan using the Destrieux parcellation, implemented in FreeSurfer (Destrieux et al., 2010).

### Definition of age-bins through sliding-window approach

**Figure 1:**
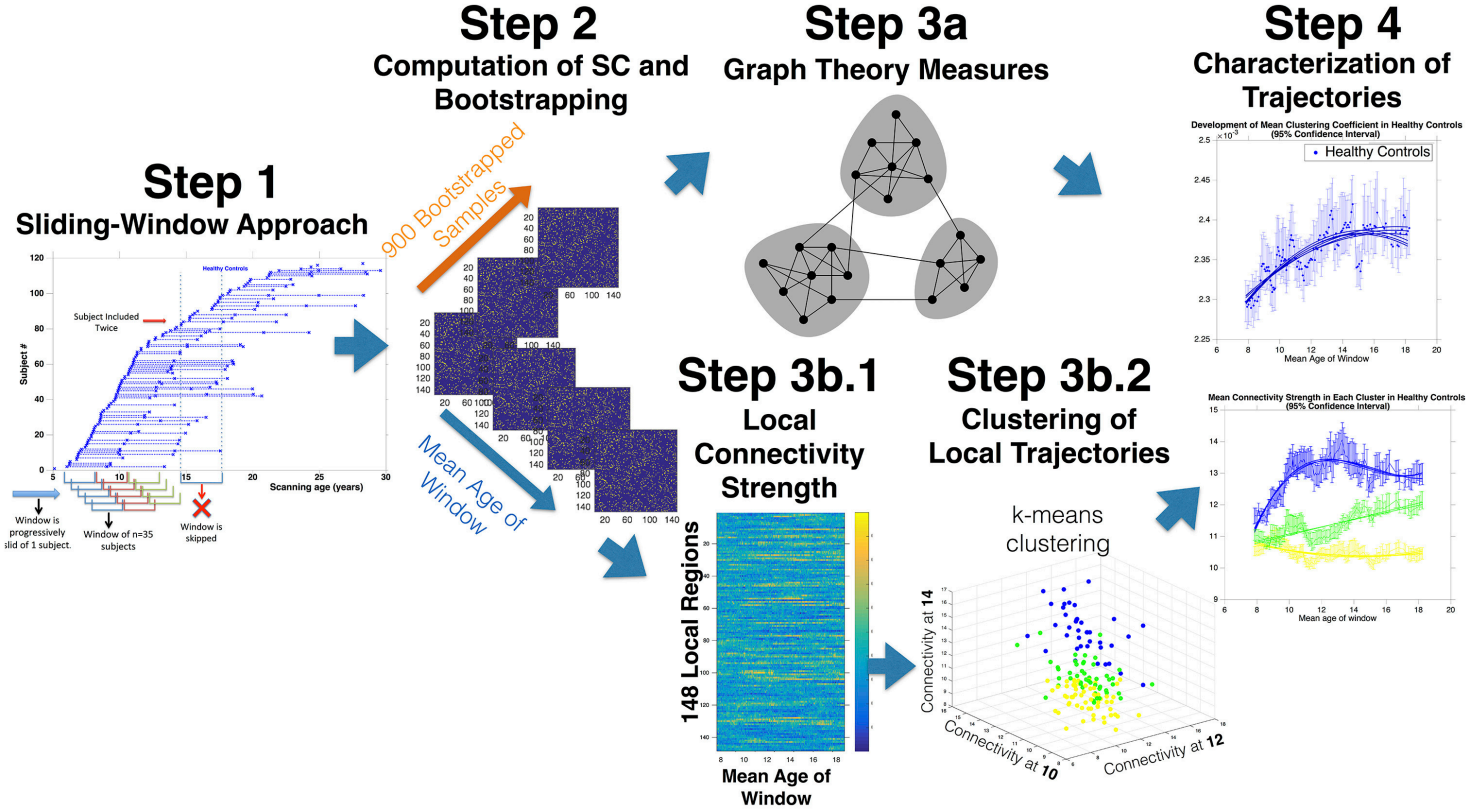
Sliding-Window Methodological Protocol: **Step 1:** Scans are ordered according to age. Subsequently a window of 35 subjects is progressively slid across the cohort starting for the 35 youngest scans at advancing by of one scan at a time. The procedure yields partially overlapping age-windows of progressively increasing mean age. **Step 2**: Covariance matrices are computed in each partially overlapping age-window. Subsequently scans included in each age-window are resampled for 900 leave-one-out substitution bootstrapped samples (BSs). **Step 3a**: The global architecture of covariance matrices computed in each age-window and each BS are characterized with Graph Theory measures, obtaining a distribution of each measure at each age-window. **Step 3b.1**: Local connectivity strength is computed for each region and each age-window yielding a matrix of regions by age. **Step 3b.2:** K-means clustering is then employed to identify clusters of regions showing common maturation. Connectivity strength is averaged within each cluster at each age-window. **Step 4**: Models of increasing order are fit to measures of SC and BIC are employed to select model order. The process is repeated for 900 BSs to define confidence intervals of developmental trajectories.

Visits were ordered according to age separately for HCs and patients. Subsequently, a window of 35 visits was progressively slid across the two cohorts, starting for the 35 visits of the youngest subjects, and proceeding with one visit at a time (See Figure 1, Step 1). Definition of window-width is an inherently arbitrary step of sliding-window approaches and implies a trade of between higher statistical power and lower temporal resolution with increasing window-width (Preti et al., 2016). We opted for a window-width of 35 subjects given that 30 data-points are generally considered sufficient for the estimation of reliable correlations (Hogg et al., 2007). Moreover a window-width of 35 subjects was associated with a mean age-range in each window of 2.86 ± 0.72 years in 22q11DS and 2.83 ± 0.72 in HCs that was only slightly inferior to the mean time between longitudinal visits of 3.8 and 3.6 years in 22q11DS and HCs respectively. This guaranteed that subjects were generally not included twice, at different time-points in a single window. Age-windows that included two repeated visits of the same subject were excluded (37 and 43 age-window in 22q11DS and HCs respectively). This yielded a total of 148 and 132 partially overlapping age-windows, of progressively increasing mean age, in 22q11DS and HCs, respectively. In the subsequent steps, SC was estimated in every age-window.

### Structural covariance estimation

Before computing SC we accounted for the effects of nuisance variables. Specifically, we controlled for the effects of scanner and gender in two steps: at the level of the entire cohort, and then again in each individual age-window. In each window, we additionally controlled for the effects of age and overall cortical thickness as commonly described in SC literature (Alexander-Bloch et al., 2013).

Subsequently, we computed SCNs using Pearson correlations of cortical thickness across subjects between each couple of brain regions, yielding a symmetrical 148 × 148 covariance matrix for each age-window.

We computed several measures to characterize SCNs after thresholding to consider only positive correlations (Figure 1, Step 3a). Mean R Coefficient across all connections was computed as an index of mean connectivity strength (MCS). MCS was also computed for each local region. We employed graph theory to quantify features of network architecture using functions implemented in the Brain Connectivity Toolbox for MATLAB (The MathWorks, Inc., Natick, MA; http://www.brain-connectivity-toolbox.net/). As graph-theoretical measures are influenced by overall network connectivity, we normalized for this by dividing each correlation for the mean of all R-coefficients (Rubinov and Sporns, 2010; van Wijk et al., 2010).

We quantified Mean Clustering Coefficient (MCC) as an architectural measure of local segregation. MCC measures the proportion of neighbors of a node that are also neighbors to each other (Rubinov and Sporns, 2010) and quantifies how efficiently information is transferred within segregated clusters of regions. We also quantified Network Efficiency (NE). NE gauges how efficiently connections are distributed to limit the distance separating each couple of regions of a network (Rubinov and Sporns, 2010).

### Extraction of SC developmental trajectory and analysis

We firstly tested the age-relationship of SC measures in both populations separately. Specifically, models of increasing order (from constant to cubic age-relationship) were fit to each measure of SC and a Bayesian information criterion (BIC) based approach was used to select the optimal model order (Mutlu et al., 2013; Figure 1, Step 4). Subsequently visits included in each age-window were randomly resampled for 900 leave-one-out-substitution Bootstrap Samples (BSs) (Figure 1, Step 2). Covariance matrices were re-computed at each BS, yielding a distribution of SC measures in each age-window. Model fitting was then repeated at each bootstrap iteration and correlation with age was considered significant if a model of non-zero order could be fit to at least 95% of the BSs. For each population we additionally computed confidence intervals (CIs) of curve parameters (such as CI of ages of peak maturation).

Subsequently we employed K-means clustering to identify clusters of regions showing common maturation (Figure 1, Step 3b.2).Indeed K-means clustering allows to group together variables (i.e., brain regions) that are similar throughout multiple dimensions (i.e., connectivity at different ages) (Bair, 2013). See Figure 1, Step 3b.2 for a graphical representation of K-means clustering approach. Here, the algorithm yielded clusters of regions whose connectivity strength was similar throughout multiple age-windows, indicating a common developmental trajectory. We tested several cluster solutions (from *k* = 2 to *k* = 7) and employed a silhouette approach to identify the optimal number of clusters (Rousseeuw, 1987). We subsequently averaged connectivity strength across regions within each cluster and employed bootstrapping to define CIs at each age-window. We then fit models of increasing order to the bootstrapped data to test and characterize developmental trajectories of each cluster.

Lastly we tested for between-group differences in developmental trajectories of SC measures. Specifically group differences were defined when either models of different order were fit to the two populations, or when the CI of peak-maturation age did not overlap. To test for quantitative differences in SC measures we identified the 120 couples of windows that were closest in terms of mean-age. We then computed *p*-values for each measure as the proportion of overlap in bootstrapping-derived distributions, between the 2 populations.

### Correlation with trajectories of working-memory

Working-Memory (WM) performance was measured using the Wechsler Digit Span subtest, backward part, considering raw-scores (Wechsler, 1991, 1997). In this task, participants were asked to repeat backward a gradually increasing set of numbers. WM was tested at each scanning session and WM scores were available for 206/211 visits in HCs and 216/221 visits in 22q11DS.

To characterize developmental trajectories of WM we firstly employed mixed model linear regression at the level of individual subjects in the two populations. Detailed description of the specific algorithm employed is available in previous work (Kremen et al., 2010). Briefly models of increasing order (from constant to cubic) were fit to WM scores and a BIC based approach was used to select optimal model order. Hence a likelihood ratio test was employed to test differences in both curve shape, also known as an interactions effect, and in curve intercept, also known as group effect. To qualitatively characterize differences in curve shape, we furthermore plotted the derivatives of mean developmental curves that express the rate of WM maturation as a function of age in the two populations.

To allow a direct comparison with SC, mean WM scores were furthermore computed in each window using the previously described sliding window approach. Mean WM was then correlated with measures of SC computed in each window using Pearson's correlation. Finally to define CIs, correlations between WM and SC were recomputed for 900 BSs. Correlations were considered significant if the null hypothesis could be rejected at *p* < 0.05 in at least 95% BSs. Moreover to exclude that age was not exclusively responsible for correlations between WM performance and SC, we repeated correlations after accounting for the effect of age.

### Correlation with severity of internalizing psychopathology in 22q11DS

Severity of internalizing psychopathology was measured with the Child Behavior Checklist (CBCL) (Achenbach, 1991), filled out by parents of patients with 22q11DS younger than 18 years of age. Patients with 22q11DS older than 18 filled out the Adult Behavior Checklist (ABCL) (Achenbach and Rescorla, 2003). To account for systematic inconsistencies across the two instruments values obtained from CBCL and ABCL were separately z-scored prior to being merged. Subsequently the same statistical procedure adopted for WM scores was employed to describe developmental trajectories of internalizing psychopathology, using both mixed models linear regression and the sliding-window approach. Finally, as described for WM, internalizing symptom scores computed from the sliding window approach were correlated with measures of SC for each of 900 BSs, before and after accounting for the effect of age. Correlations were considered significant if the null hypothesis could be rejected at *p* < 0.05 in at least 95% BSs.

## Results

### Developmental trajectories of mean connectivity strength

**Figure 2:**
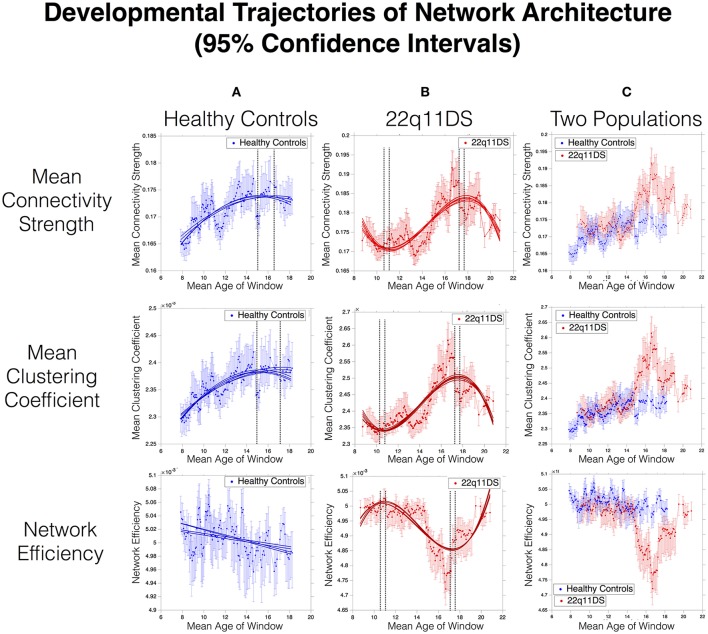
Developmental trajectories of network architecture in HC **(A)** and 22q11DS **(B)**. Dashed lines indicate 95% confidence intervals of ages of peak maturation. **(C)** displays the overlap in developmental trajectories between the two populations. Lack of overlap in 95% confidence intervals indicates a statistically significant difference at *p* < 0.05. Precise *p*- values are computed as the proportion of overlap in bootstrapped derived distributions and are displayed in Supplementary Figure 3.

Mean network connectivity strength in HCs showed a significant quadratic developmental curve with peak maturation occurring at [15.1–16.5] Years of Age (YoA). In 22q11DS, **MCS** showed an altered cubic development with a first negative peak at [10.9–11.2] YoA, followed by a second positive peak at [17.7–17.9] YoA. Overlapping developmental curves between patients and controls revealed a significantly higher mean connectivity in 22q11DS for 29 age-windows, mostly (23/29) located between 14.8 and 18.1 YoA (See Figure 2).

### Developmental trajectories of network architecture

MCC in HCs showed a quadratic trajectory of development peaking at [15.2–17.3] YoA. In 22q11DS, MCC showed a deviant cubic development with a first negative peak at [10.35–10.78] YoA, followed by a positive peak at [17.5–17.71] YoA. Direct comparison of two curves revealed a significantly higher MCC in 22q11DS for 22 age-windows spanning between 14.8 and 18.15 YoA (See Figure 1).

In HCs, NE showed a linear decrease throughout the age range. In 22q11DS, maturation of NE was altered and was best captured by a cubic trajectory with a first negative peak at [10.69–10.99] YoA, followed by a positive peak at [17.25–17.42] YoA. Overlapping the two curves revealed that NE was significantly lower in 22q11DS (*p* < 0.05) for 30 age-windows mostly (25 out of 30) located between 14.9 and 18 YoA (See Figure 1).

### Clustering of regions according to developmental trajectories of connectivity strength

**Figure 3:**
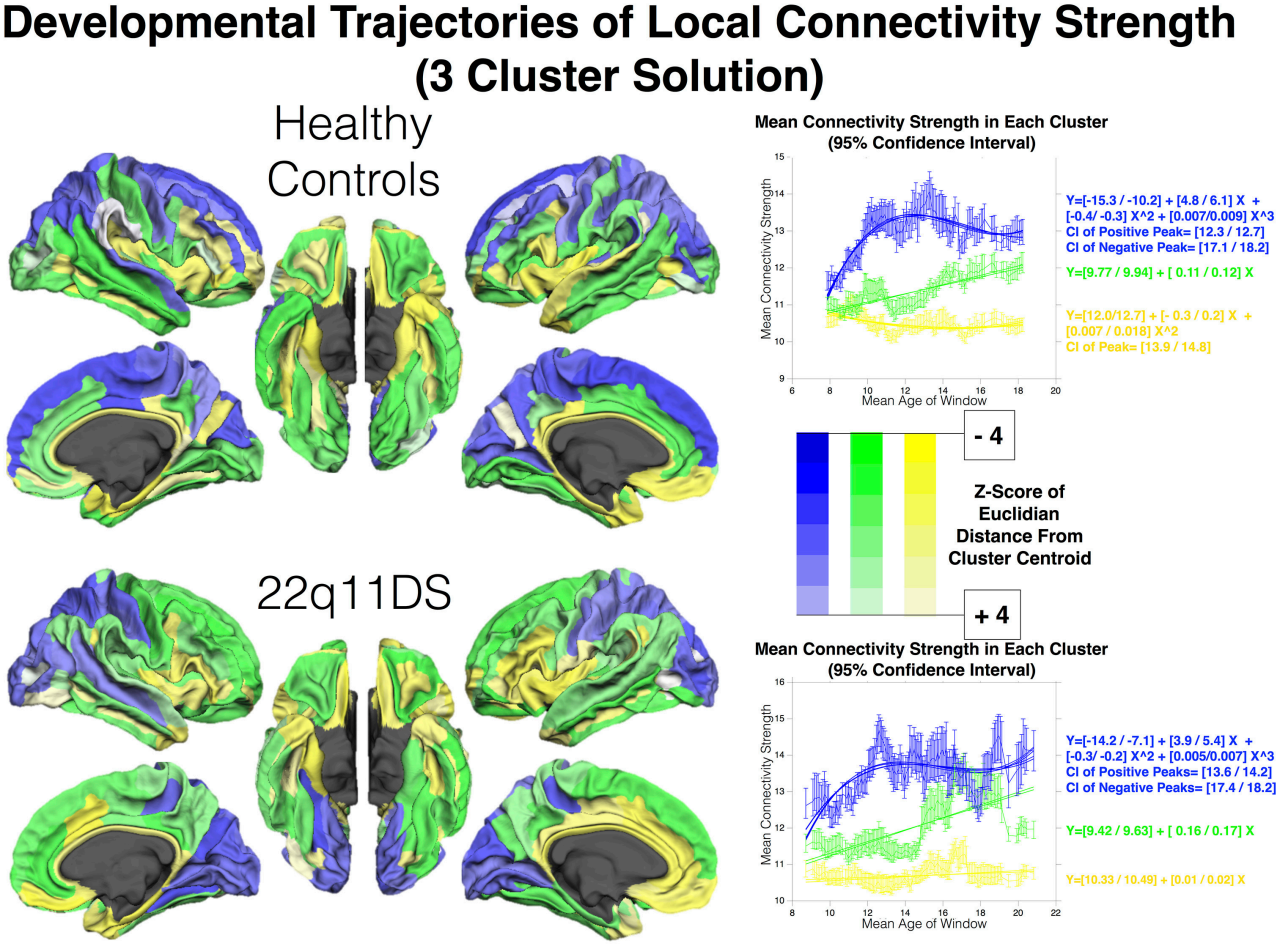
Clustering of regions according to developmental trajectories of connectivity strength in HCs and patients. Color-coding (green, blue, and yellow) indicates correspondence between cluster and developmental trajectory. Regions are shaded according to Z-score of mean Euclidian distance from cluster centroid computed over 900 bootstrapped samples, which is indicative of how closely maturation of each region is reflected in that of the corresponding cluster.

The silhouette approach identified *K* = 2 and *K* = 3 as the two best cluster solutions for both populations. Here we describe results for the 3-cluster solution, that best characterized differences between populations (See Figure 3). Results for the 2-cluster solution are reported in Supplementary Figure 2.

In HCs, a first dominant cluster presented a cubic developmental trajectory, with a first positive peak at [12.34–12.73] YoA, followed by a negative peak at [17.12–18.25]. This first cluster encompassed mostly bilateral fronto-parietal regions including bilateral middle and superior frontal gyri and pre-central gyrus, inferior and superior parietal gyrus, the precuneus, cunes, and superior occipital gyrus.

A second cluster of regions showed a more retarded and protracted linear maturation and included the bilateral middle and inferior temporal gyri, fusiform cortices, anterior cingulate cortex (ACC), orbito-frontal cortex (OFC), sub-parietal sulcus, and left inferior parietal lobule.

A third cluster of regions, that was characterized by the weakest connectivity strength, encompassed bilateral insular cortices, parahippocampal giri, posterior cingulate gyri and pericallous gyrus and presented a subtle quadratic development with a negative peaking at [13.98–14.83] YoA.

In 22q11DS, the 3-cluster solution revealed a strikingly different developmental pattern.

A first cluster showed a cubic development with a first positive peak at [13.63–14.28] YoA, followed by a second negative peak at [17.41–18.26] YoA. This trajectory is similar to the one observed in HCs. However, this first cluster included exclusively parietal and occipital regions such as bilateral middle and superior occipital gyri, occipital poles, cuneus, inferior and superior parietal gyri and post-central gyri.

Frontal regions, on the other hand, were grouped with a second cluster that showed a more postponed and protracted linear development. This second cluster encompassed the bilateral middle and superior frontal gyri, precentral gyrus, along with several regions that showed a comparable developmental trajectory in HCs, such as the middle and inferior temporal gyri, fusiform cortex, OFC, parietal lobule and right ACC.

Lastly, a third cluster of regions was characterized by weaker connectivity strength along with a subtle quadratic development throughout the age range. This last cluster encompassed the insular cortex, inferior frontal gyrus, gyrus rectus, para-hippocampal gyrus, left anterior and posterior cingulate cortex and left superior temporal gyrus.

### Developmental trajectories of working memory performance

**Figure 4:**
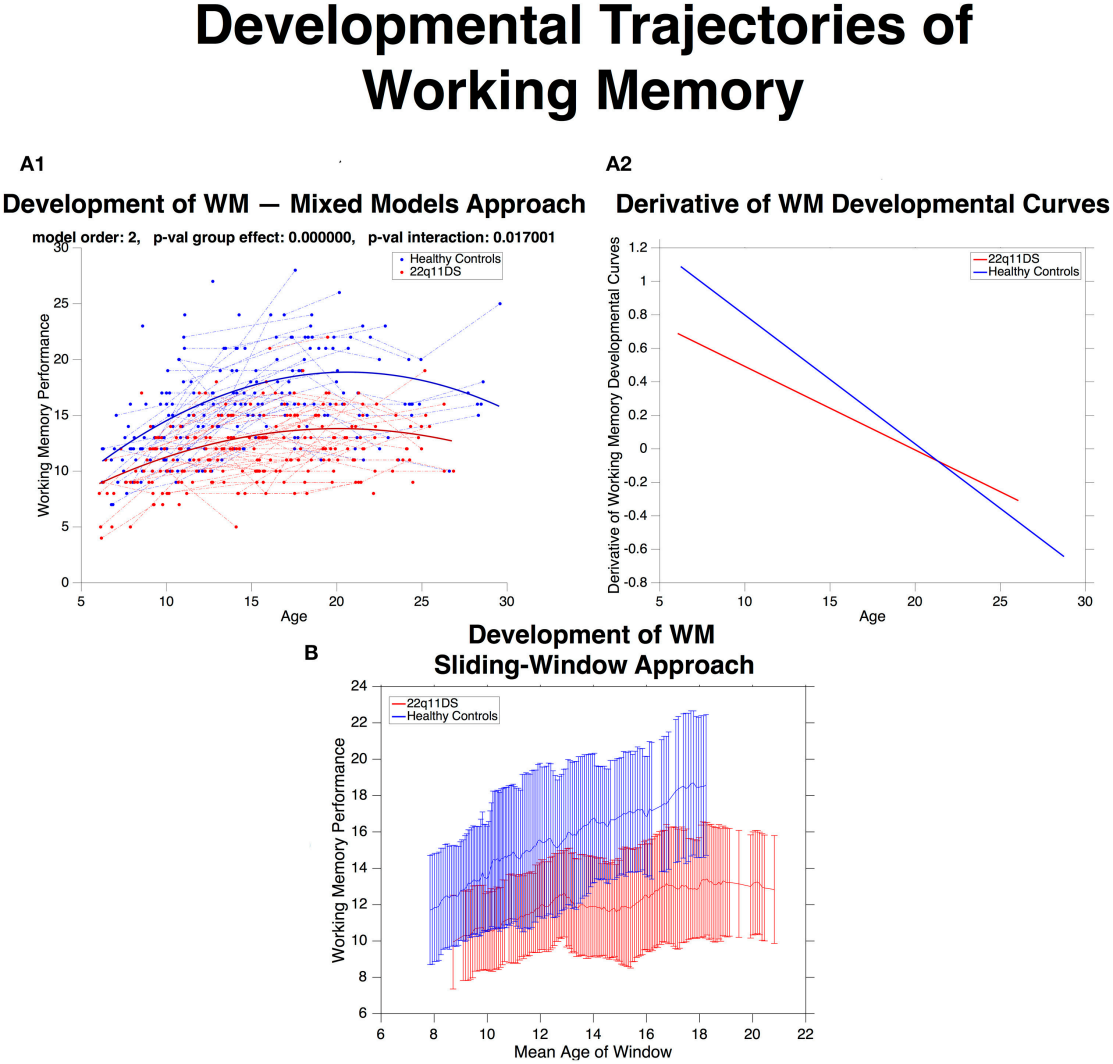
**(A.1)** Developmental trajectories of working memory (WM) described using mixed-model linear regression for HCs in blue and 22q11DS in red. WM is on average lower in 22q11DS (*p*-val group effect < 0.0001) and undergoes aberrant development with age (*p*-val interaction = 0.01). **(A.2)** Derivatives of WM developmental curves, express mean rate of WM maturation as a function of age for HCs in blue and 22q11DS in red. Strongest differences in rate of WM development are observable at the youngest ages, during late-childhood and early adolescence while by late adolescence rate WM maturation is similar between the two populations. **(B)** Developmental trajectories WM are estimated using the same sliding-window approach used to compute structural covariance. Error-bars (HCs in blue and 22q11DS in red) indicate mean ± standard deviation of WM scores in each window.

Overall WM performance was significantly lower in 22q11DS compared to HCs, as estimated by a significant group effect (*p* < 0.0001). WM underwent a quadratic developmental trajectory in both populations with the strongest increase occurring in late childhood and early adolescence (See Figure 4). However the shape of the trajectory was significantly different in 22q11DS (p of interaction = 0.01). Inspection of the derivatives of mean developmental trajectories revealed that rate of WM development was significantly reduced in 22q11DS particularly during late childhood and early adolescence. By late-adolescence/early adulthood rate of WM development was similar across the two populations. A similar picture was depicted when estimating developmental trajectories with the sliding-widow approach (See Figure 4).

### Correlation of working memory and network architecture

**Figure 5:**
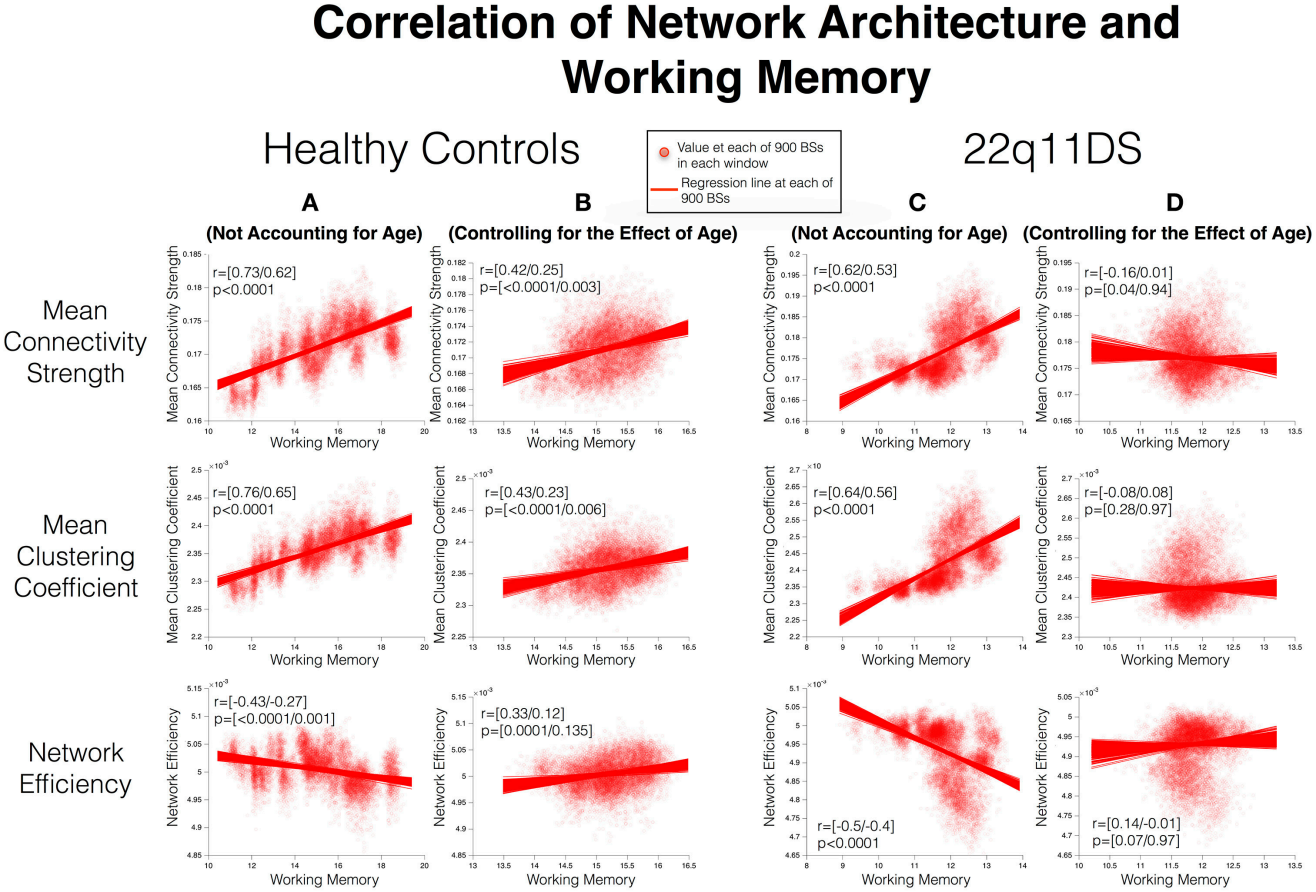
Correlation between structural covariance network architecture and working memory. Method: Mean WM scores and measures of SC network architecture are computed in each window and correlated using Pearson's correlation. The process is repeated for 900 BSs in each window to define confidence intervals of correlations. Correlations are furthermore repeated after controlling for the effect of age. **(A)** Correlations in HCs before controlling for age. **(B)** Correlations in HCs after controlling for age. **(C)** Correlations in 22q11DS before controlling for age. **(D)** Correlations in 22q11DS after controlling for age.

In HCs before accounting for the effect of age, WM performance was positively correlated with **MCC** (*r* = [0.73/0.62] *p* < 0.001) and **MCS** (*r* = [0.73/0.62], *p* < 0.001) whereas it was negatively correlated with **NE** (*r* = [−0.43/−0.27], *p* = [<0.0001/0.001]). After accounting for the effect of age, WM remained positively correlated with **MCC** (*r* = [0.43/0.23], *p* = [<0.0001/0.006]) and **MCS** (*r* = [0.42/0.25], *p* = [<0.0001/0.003]) whereas correlation with **NE** became positive and non-significant (*r* = [0.33/0.12], *p* = [0.0001/0.135]) (See Figure 5).

In 22q11DS **MCC** (*r* = [0.64/0.56] *p* < 0.0001) and **MCS** (*r* = [0.62/0.53] *p* < 0.0001) were positively correlated while NE was negatively correlated (*r* = [−0.5/−0.4] *p* < 0.0001) with WM before accounting for the effect of age. However after accounting for the effect of age WM was not significantly correlated with either **MCC** (*r* = [−0.08/0.08] *p* = [0.28/0.97), **MCS** (*r* = [−0.16/0.01] *p* = [0.04/0.94]) or NE (*r* = [0.14/−0.01] *p* = [0.07/0.97]) in 22q11DS.

### Correlation of working memory and local connectivity strength

**Figure 6:**
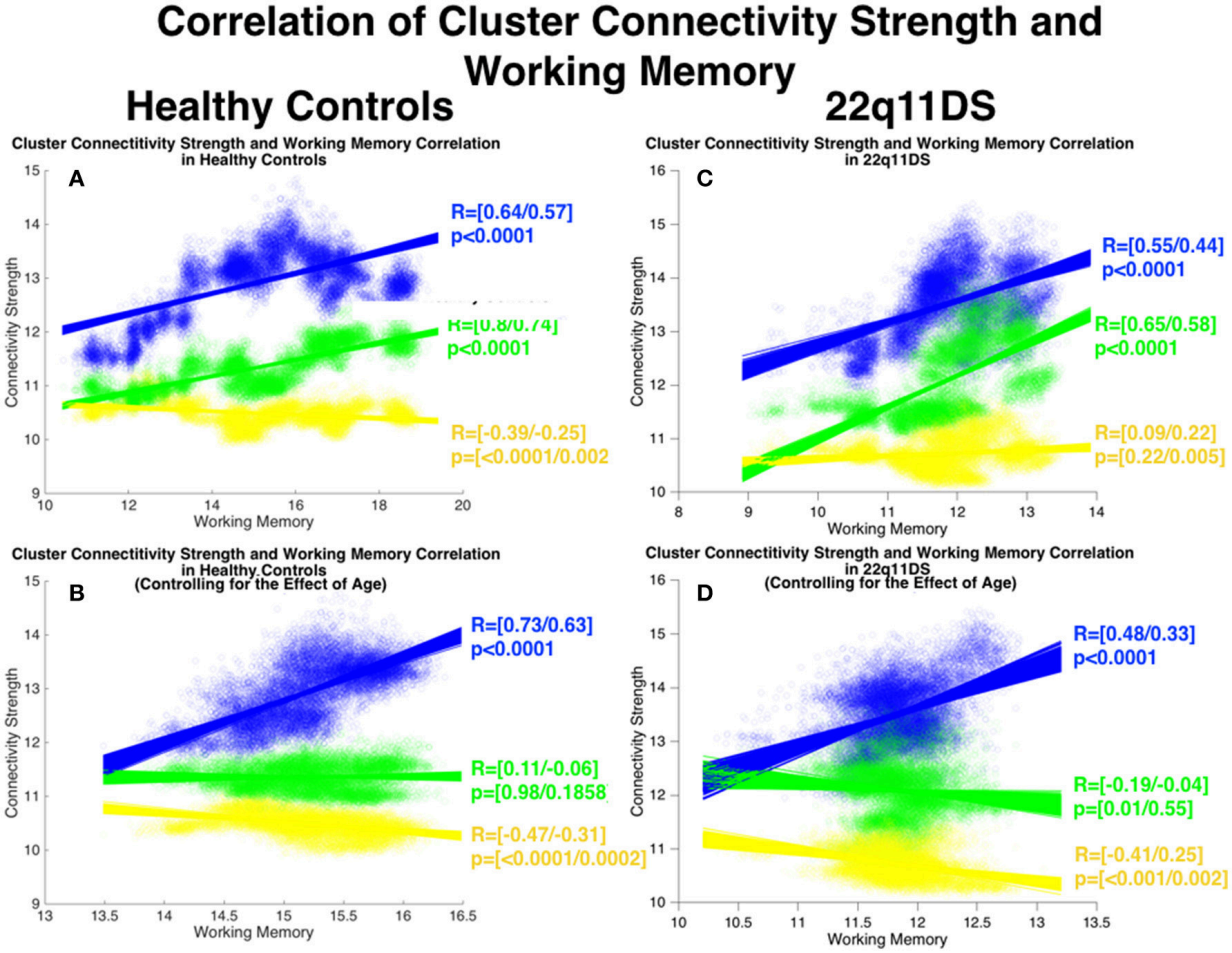
Correlation between structural covariance local connectivity strength and working memory. Method: Mean connectivity strength in each cluster and mean WM scores are computed in each window and are correlated using Pearson's correlation. The process is repeated for 900 BSs in each window to define confidence intervals of correlations. Correlations are furthermore repeated after controlling for the effect of age. **(A)** Correlations in HCs before controlling for age. **(B)** Correlations in HCs after controlling for age. **(C)** Correlations in 22q11DS before controlling for age. **(D)** Correlations in 22q11DS after controlling for age.

As pertains to local connectivity strength in HCs, connectivity of the (blue) fronto-parietal cluster (*R* = [0.64/0.57] *p* < 0.0001) and the (green) ACC-OFC cluster (*R* = [0.8/0.74] *p* < 0.0001) were positively correlated with WM whereas connectivity of the yellow cluster was negatively correlated with WM (*R* = [−0.39/−0.25] *p* = [<0.0001/0.0027]) (See Figure 6). However after accounting for the effect of age only the blue fronto-parietal cluster remained positively correlated with WM (*R* = [0.73/0.63] *p* < 0.0001) while no correlation was observed for the green cluster (*R* = [0.11/−0.06] *p* = [0.98/0.1858]) and a negative correlation was observed for the yellow cluster (*R* = [−0.47/−0.31] *p* = [<0.0001/0.00).

In 22q11DS connectivity of the blue (*R* = [0.55/0.44] *p* < 0.0001) and the green (*R* = [0.65/0.58] *p* < 0.0001) clusters were positively correlated with WM whereas no significant correlation was observed for the yellow cluster (*R* = [0.09/0.22] *p* = [0.22/0.005]). After accounting for the effect of age only the blue parito-occipital cluster remained positively correlated with WM (*R* = [0.48/0.33] *p* < 0.0001) whereas no significant correlation was observed for the green cluster (*R* = [−0.19/−0.04] *p* = [0.01/0.55]) and a negative correlation was observed for the yellow cluster (*R* = [−0.41/0.25] *p* = [<0.001/0.002]).

### Correlation of SC and severity of internalizing symptoms in 22q11DS

**Figure 7:**
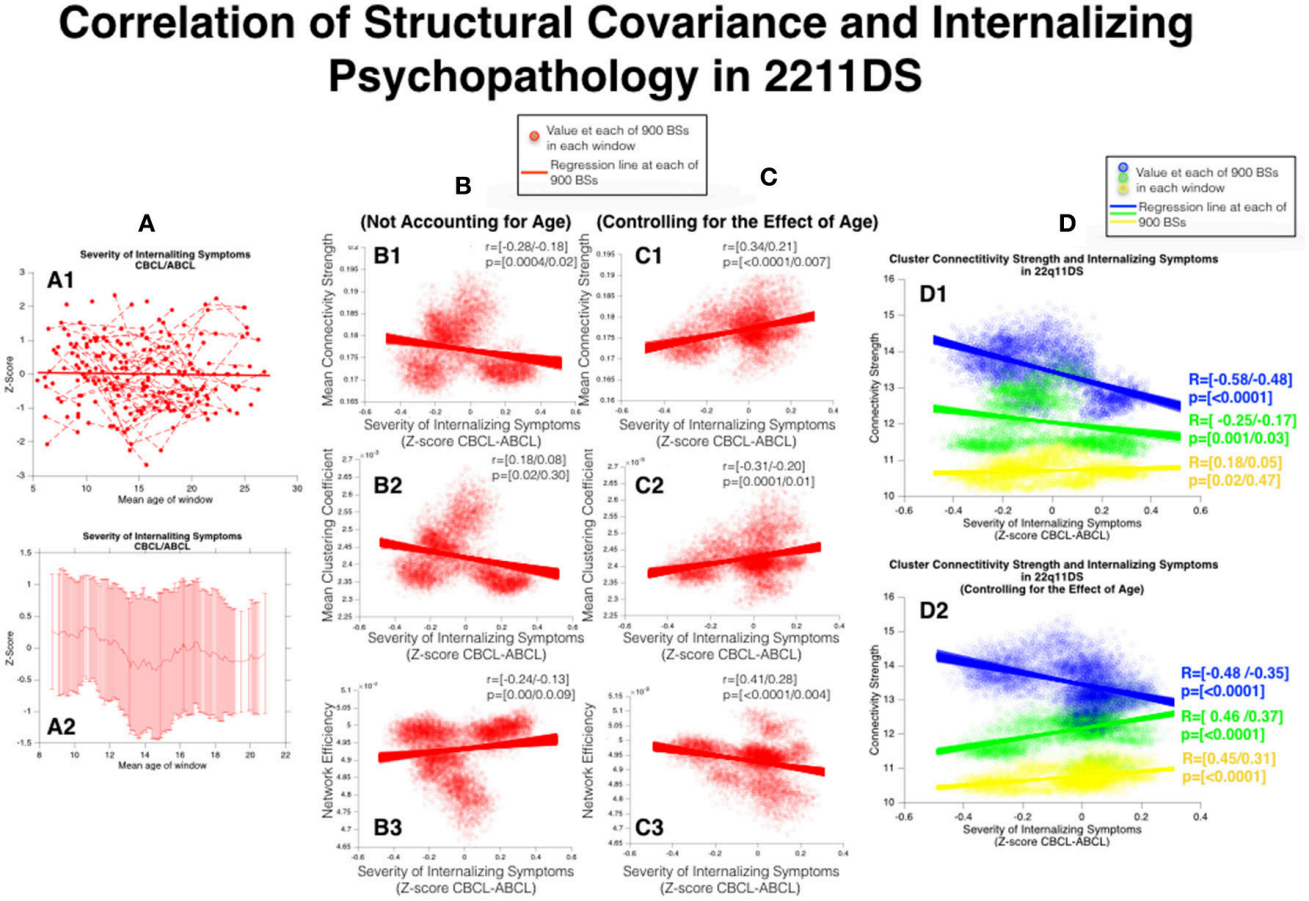
Correlation of structural covariance and internalizing psychopathology in 22q11DS. **(A)** Developmental trajectories of internalizing symptoms as quantified from the CBCL/ABCL subscale. Measures obtained from the two instruments are separately z-scored prior to being merged. **(A1)** Mixed models linear regression approach **(A2)** Sliding Window Approach. **(B)** Correlation of internalizing psychopathology and network architecture before accounting for the effect of age **(B1)** MCS, **(B2)** MCC, **(B3)** MCS. **(C)** Correlation of internalizing psychopathology and network architecture after accounting for the effect of age **(C1)** MCS, **(C2)** MCC, **(C3)** MCS. **(D)** Correlation of structural covariance local connectivity strength and internalizing psychopathology **(D1)** Before accounting for the effect of age **(D2)** After accounting for the effect of age.

According to mixed linear regression severity of internalizing symptoms remained stable with age in 22q11DS. However the sliding window approach revealed a more complex pattern with reduced symptom severity during childhood and a transient increase in internalizing symptom severity during mid to late adolescence (See Figure 7, Column **A**).

Before accounting for the effect of age severity of internalizing symptoms was negatively correlated with **MCC** (*r* = [−0.28/−0.18], *p* = [0.0004/0.02]) whereas no significant correlation was observed for NE (*r* = [0.18/0.08], *p* = [0.02/0.3]) or for **MCS** (*r* = [−0.24/−0.13], *p* = [0.001/0.09]). However after accounting for the effect of age severity of internalizing symptoms was positively correlated with both **MCC** (*r* = [0.34/0.21], *p* = [<0.0001/0.007]) and **MCS** (*r* = [0.41/0.28], *p* = [<0.0001/0.004]) and negatively correlated with NE (*r* = [−0.31–/0.20], *p* = [<0.0001/0.01]) (See Figure 7, Column **B,C**).

As pertains to local connectivity strength, before accounting for the effect of age, internalizing symptom severity was negatively correlated with connectivity of both the blue parieto-occipital cluster (*r* = [−0.58/−0.48], *p* < 0.0001) and green cluster (*r* = [−0.25/−0.17], *p* = [0.001/0.03]) whereas no significant correlation was observed for the yellow cluster (*r* = [0.18/0.05], *p* = [0.02/0.47]). However after accounting for the effect of age internalizing symptom severity remained negatively correlated with connectivity of the blue parieto-occipital cluster (*r* = [−0.48/−0.35], *p* < 0.0001) but was positively correlated with connectivity of both the green (*r* = [0.46/0.37], *p* < 0.0001) and yellow (*r* = [0.45/0.31], *p* < 0.0001) clusters (See Figure 7, Column **D**).

## Discussion

In this work, we implement a state-of-the are sliding window approach to investigate developmental trajectories of SC networks in a large cohort of patients with 22q11DS and HCs.

We will first discuss findings in HCs, in relation to typical trajectories of cognitive maturation and offering hypotheses on the neurobiological processes underlying SC maturation. We will then discuss how this development deviates in patients with 22q11DS. We advance that disturbed SC maturation may contribute to core developmental features of the syndrome, including disturbed cognitive maturation during childhood and internalizing psychopathology and psychosis predisposition during adolescence.

### Maturation of structural covariance network architecture in healthy controls

Our results in HCs point to late-childhood and early-adolescence as critical periods for the maturation of SCNs. Indeed, until early-adolescence, networks underwent a significant increase in mean correlation strength along with a prominent increase of MCC, indicative of a more segregated and less random organization. Architectural maturation was also reflected by a decrease in NE, that was however more gradual throughout the examined age range. Late-childhood re-organization of SCN architecture was previously reported in a large cross-sectional cohort (Khundrakpam et al., 2013). Our results firstly replicate this finding in a longitudinal sample, furthermore allowing a more precise characterization of developmental trajectories.

From the perspective of cortical development, the period of late-childhood has until recently been relatively overlooked, mainly due to histological studies reporting little changes in neuronal or synaptic organization during this time period (Huttenlocher, 1979; Rakic et al., 1986; Huttenlocher and Dabholkar, 1997; Petanjek et al., 2011). Indeed synaptogenesis is completed by early-childhood while most synaptic pruning occurs after the onset of adolescence (Huttenlocher, 1979; Rakic et al., 1986; Huttenlocher and Dabholkar, 1997; Petanjek et al., 2011). However, late-childhood is a period of important maturation for multiple cognitive domains, including verbal and non-verbal intelligence, attentional performance and executive functions (Chelune and Baer, 1986; Anderson, 2002; Crone et al., 2006). A possible interpretation for this discrepancy is that late-childhood may be a critical period for synaptic fine-tuning (Changeux and Danchin, 1976). During synaptic fine-tuning, functionally relevant synapses are stabilized in order to be protected from subsequent pruning during adolescence. Interestingly, it was proposed that late-childhood maturation of SCNs could capture this process of synaptic fine-tuning (Khundrakpam et al., 2013), which is critical for cognitive maturation.

An alternative interpretation is that the reorganization of SCNs is reflecting white matter maturation. Indeed, developmental trajectories of white matter from childhood to adulthood are best described by quadratic curves, with most of the development occurring precociously, during childhood and early adolescence (Lebel and Beaulieu, 2011; Lebel et al., 2012). Furthermore SC has shown considerable overlap with white matter connectivity (Sui et al., 2014).

Importantly SC network architecture was correlated with WM performance suggesting a potential functional relevance of SC maturation. A more segregated and organized SC network architecture has been previously associated with higher cognitive performance in childhood and adolescence (Khundrakpam et al., 2016). When considering that in HCs network segregation significantly increases during childhood and early adolescence, correlation between MCC and WM suggests that maturation of network architecture could contribute to cognitive development during this critical developmental period. On the other hand NE underwent a linear decrease with age, but showed a non-significant positive correlation with WM when the effect age was accounted for. Recent findings pointed to a positive correlation between cognitive performance and SC NE (Khundrakpam et al., 2016). Our results, albeit at trend level, tend to confirm that higher SC NE is associated with better WM irrespective of age. However, when considering developmental trajectories, our results suggest that maturation of NE is not strongly implicated in development of WM, at least in the examined age range.

### Deviant maturation of structural covariance network architecture in 22q11DS

Trajectories of SC network architecture were altered in 22q11DS with a lack of development during childhood, followed by a prominent reorganization during adolescence. Network development was not only postponed, but also aberrant with patients presenting increased connectivity strength coupled with excessive segregation and insufficient integration, for several age-windows, during mid-to-late adolescence.

In a previous cross-sectional investigation of SC in 22q11DS, we reported increased correlation strength, coupled with decreased architectural integration and increased segregation, that selectively affected patients presenting prodromal psychotic symptoms both compared to HCs and non-psychotic patients (Sandini et al., 2017). Several studies investigating SC in patients suffering from psychotic symptoms have also reported increased correlation strength (Wible et al., 1995, 2001; Buchanan et al., 2004; Mitelman et al., 2005a,b, 2006; Modinos et al., 2009; Zugman et al., 2015), along with increased architectural segregation and decreased integration (Bassett et al., 2008; Zhang et al., 2012). Increased segregation and decreased integration are furthermore consistent with reports of white-matter dysconnectivity in idiopathic psychosis (van den Heuvel and Fornito, 2014) and in 22q11DS (Ottet et al., 2013b; Váša et al., 2016) as well as with findings of increasingly segregated functional networks in 22q11DS (Scariati et al., 2016b).

Here disturbed network architecture was correlated with higher internalizing symptoms after accounting for the effect of age. High internalizing psychopathology, including anxiety, depression, social withdraw, and thought disorders represents a hallmark of psychiatric phenotype of 22q11DS (Shashi et al., 2012; Klaassen et al., 2013, 2015). Moreover internalizing symptoms such as anxiety, exert a prominent negative impact on overall functioning (Angkustsiri et al., 2012) and represent a strong risk factor for the subsequent development of psychosis in 22q11DS (Gothelf et al., 2007, 2013). Internalizing symptoms have furthermore been shown to increase in prevalence during adolescence in 22q11DS (Duijff et al., 2013). Our finding suggest that the development of disturbed network architecture, with insufficient integration and excessive segregation, could contribute to the emergence of internalizing symptoms and potentially to increased vulnerability to psychosis during adolescence in 22q11DS. Indeed disturbed architecture could lead to an insufficient integration of signals originating from functionally specialized sub-networks, which could in turn predispose to the emergence of the syndrome's psychiatric phenotype (Sandini et al., 2017).

A second observation is that the aberrant development of network architecture during adolescence is preceded by a lack of typical maturation during late-childhood. As discussed in the previous section, late-childhood is the critical period for the maturation of SCNs in HCs (Khundrakpam et al., 2013) and development of particularly MCC could contribute to improved WM in this period. The lack of architectural development during childhood in 22q11DS could therefore contribute to the blunted WM maturation observed in this population. However measures of SC network architecture were not significantly correlated with WM performance in 22q11DS, after accounting for the effect of age.

From the perspective of the underlying neurobiology, if SCNs maturation were indeed capturing a process of synaptic stabilization (Changeux and Danchin, 1976), insufficient late-childhood network maturation in 22q11DS could reflect impairments in this process. Interestingly, synaptic instability has been reported in LGDel± mouse models of 22q11DS (Moutin et al., 2016). A similar deficit of synaptic stabilization might therefore also be affecting patients with 22q11DS, and manifest with a lack of network reorganization observed during late-childhood. Synaptic instability could then potentially predispose to aberrant SC development and vulnerability to internalizing psychopathology and psychosis during adolescence. Indeed, recent neuro-pathological evidence has suggested that synaptic deficits, that are highly replicable in schizophrenia (Garey et al., 1998; Glantz and Lewis, 2000; Rosoklija et al., 2000; Black et al., 2004; Glausier and Lewis, 2013), might be linked to insufficient synaptic stabilization (MacDonald et al., 2017).

Moreover disturbed SC maturation during late-childhood could also be reflective of deficient white-matter maturation, that might contribute to typical development of SC in HCs. Although several cross-sectional investigations have consistently reported white matter dysconnectivity in 22q11DS (Ottet et al., 2013a,b; Scariati et al., 2016a; Váša et al., 2016) so far no longitudinal studies have been conducted. Our results could suggest that developmental trajectories of white-matter connectivity start to diverge during late-childhood potentially inducing vulnerability to the emergence of psychosis during adolescence.

### Developmental trajectories of local connectivity strength in healthy controls

In HCs, a first set of regions displayed a precocious cubic maturation, with increase in connectivity up to late-childhood, followed by a more subtle pruning during adolescence and a stabilization by early adulthood. Recent work, employing a similar sliding window approach, reported comparable trajectories for SC connectivity maturation for regions corresponding to this first cluster, albeit limited to the age range between late adolescence to early adulthood. From a functional perspective this first cluster was mainly composed of frontal-parietal regions, strongly resembling the Central Executive Network (CEN) (Seeley et al., 2007; Menon, 2011). The CEN is critical for goal-directed cognitive processes also known as executive functions (EFs) (Wager and Smith, 2003; Fan et al., 2005; Müller and Knight, 2006; Markett et al., 2014; McKenna et al., 2017). EFs, and particularly (WM), undergo dramatic improvements during late-childhood as described both in our cohort (See Figure 4) and previous literature (Chelune and Baer, 1986; Crone et al., 2006; Tamnes et al., 2013; Ullman et al., 2014). Moreover morphological and connectivity maturation of the CEN have been specifically correlated with the development of WM (Crone et al., 2006; Tamnes et al., 2013; Ullman et al., 2014). In accord with these findings, we observe a significant positive correlation between SC connectivity of the CEN and WM performance, even when accounting for the effect of age. When considering developmental trajectories, our findings suggest that SC maturation of the CEN might contribute to the critical **WM** improvements observed during late-childhood in HCs.

A second bilateral group of regions showed a more postponed and protracted linear maturation. Connectivity of this cluster was not significantly correlated with WM performance after accounting for the effect of age. However, ACC and OFC, included in this cluster, are critical for cognitive processes underlying decision-making (Wallis and Kennerley, 2011; Khani et al., 2015) that continue to mature into early-adulthood (Blakemore and Robbins, 2012). Furthermore the inferior temporal cortices together with the ACC, sub-parietal sulcus, and inferior parietal lobule are key nodes of the default-mode-network (DMN) (Lee et al., 2013). The DMN is involved in self-referential cognitive processes (Raichle, 2015) that also continue to mature throughout adolescence (Dumontheil et al., 2010). Moreover white-matter tracts connecting most of these regions such as the cingulate bundle, or the uncinate fasciculus, continue to mature until early-adulthood (Lebel and Beaulieu, 2011), potentially explaining the delayed development of SC.

On the other hand the Salience Network, involved in the attributing subjective salience to internal and external events and classically encompassing the dorsal anterior cingulate cortex (dACC) and anterior insula (AI)(Uddin, 2015), did not appear to display a coherent developmental trajectory in HCs. Indeed while the dACC displayed a linear increase in connectivity strength, together with other DMN related regions, the AI presented a different and more subtle negative quadratic development.

### Deviant developmental trajectories of local connectivity strength in 22q11DS

Developmental trajectories of local connectivity strength were altered in 22q11DS. Indeed, compared to controls, the cluster showing a more precocious maturation included exclusively bilateral parietal and occipital regions. Frontal regions, on the contrary, were mostly included in a second cluster showing no maturation during childhood followed by increased connectivity strength during mid-to-late adolescence.

Frontal cortical dysmaturation was previously reported by our group in 22q11DS (Schaer et al., 2009). Indeed individuals with 22q11DS were found to undergo a lack of typical cortical maturation during childhood, leading to excessive cortical thickness, which was followed by accelerated cortical thinning during adolescence (Schaer et al., 2009). This pattern was particularly striking at the level of the pre-frontal cortex (Schaer et al., 2009). Our findings are therefore consistent with notion of frontal dysmaturation in 22q11DS, but expand it to consider also frontal connectivity.

As discussed in the previous section, morphological and connectivity maturation of the CEN sustain improvements in EFs and **WM**, which are particularly important during late-childhood (Crone et al., 2006; Tamnes et al., 2013; Ullman et al., 2014). Disturbed frontal connectivity maturation might therefore contribute to retarded maturation of EFs and **WM**, occurring during late-childhood in 22q11DS (Maeder et al., 2016).

Moreover in 22q11DS WM was positively correlated selectively with parieto-occipital connectivity, after accounting for the effect of age. This could suggest that in 22q11DS, parieto-occipital connectivity maturation might sustain development of **WM** during childhood, and at least partially compensate for blunted frontal maturation. Indeed two studies suggested that children and adolescents with 22q11DS might rely on more parietal-dependent cognitive strategies, with a conserved parietal activation compared to reduced frontal activation during a similar n-back non-spatial WM task (Kates et al., 2007). Moreover adolescents with 22q11DS were found to present an excessive parietal activation during an arithmetical-reasoning task compared to healthy controls (Eliez et al., 2001). However conflicting evidence has also been reported with children and adolescents presenting reduced parietal activation during a visuo-spatial WM task (Azuma et al., 2009).

A further consideration is that the prominent increase of frontal connectivity during mid to late adolescence, coincides with the emergence of disturbed network architecture. Connectivity the green fronto-temporal cluster was also positively correlated with severity of internalizing psychopathology. The increase in frontal-temporal connectivity could therefore contribute to the development of architectural disturbances, and corresponding emergence of internalizing psychopathology and vulnerability to psychosis during adolescence. Indeed several regions displaying a postponed maturation, such as the right superior frontal gyrus and right ACC were previously found to presented aberrant connectivity, selectively in patients with 22q11DS with psychotic symptoms (Sandini et al., 2017).

Moreover severity of internalizing symptoms was also positively correlated with connectivity of the yellow cluster encompassing bilateral dACC, AI and fronto-opercular cortex, that represent key regions of the Salience Network (SN) (Uddin, 2015). Disturbances in the process of salience attribution, critically governed by the SN, have been highly implicated in the pathogenesis of psychosis (Kapur, 2003). Moreover recent evidence suggests that increased connectivity of the SN might also be correlated with higher internalizing psychopathology in the peripubertal age-range (Ordaz et al., 2017). Our findings would suggest that higher connectivity of the SN might indeed contribute to increased vulnerability to internalizing psychopathology in 22q11DS, possibly as a consequence of aberrant salience attribution.

Lastly it could be tentatively hypothesized that the lack of frontal maturation during late-childhood might predispose to the subsequent aberrant frontal maturation and emergence of internalizing psychopathology and psychosis vulnerability during adolescence. Indirect support comes from the observation that a decline of frontally mediated cognitive performance, occurring as early as childhood (Kremen et al., 2010; Gur et al., 2014), is a well-documented predictor of psychosis both in 22q11DS (Vorstman et al., 2015) and in the general populations (Riecher-Rössler et al., 2009; Seidman et al., 2016).

## Conclusions

Our findings highlight critical late-childhood maturation of SCNs in HCs that could be instrumental to the significant cognitive maturation occurring during this developmental phase.

In 22q11DS, we observe disturbed development of SCNs with aberrant architecture emerging during adolescence and being preceded by a lack of typical late-childhood maturation. Disturbed development was furthermore particularly striking for frontal lobe connectivity. These results are the first to demonstrate aberrant longitudinal connectivity maturation in 22q11DS, reinforcing the hypothesis that psychosis originates from a neurodevelopmental disorder of connectivity.

## Limitations

The present work comes with one main limitation. Indeed, SC is an inherently population based measure that cannot be computed at the individual level. This consideration strongly limits direct correlations with individual cognitive and clinical variables. More advanced analysis will be required to confirm the functional relevance of our findings at level of individual subjects.

## Ethics statement

In agreement with Frontiers in Neuroscience guidelines, all participants have given their informed consent, in accordance with protocols approved by the Institutional Review Board of the University of Geneva Medical School.

## Author contributions

CS: Performed conceived the study, performed statistical analysis and redacted a first draft of the manuscript; DZ and MarS: Contributed in development of methodological pipeline, in interpretation of results and in drafting the manuscript; ES: Contributed in conceiving the study and in drafting the manuscript; MP and MauS: Contributed in drafting the manuscript and in interpretation of results; DV: Co-supervised the study, contributed in development of methodological pipeline, in interpretation of results and in drafting the manuscript; SE: Co-supervised the study, obtained funding, contributed in development of methodological pipeline, in interpretation of results and in drafting the manuscript.

### Conflict of interest statement

The authors declare that the research was conducted in the absence of any commercial or financial relationships that could be construed as a potential conflict of interest.

## Supplementary material

Supplementary Table 1 Demographic characteristics of sample.

Supplementary Table 2 Prevalence of main psychiatric and neurological diagnoses in 22q11DS sample.

Supplementary Figure 1 Age distribution of sample.

Supplementary Figure 2 Developmental trajectories of local connectivity strength (two cluster solution).

Supplementary Figure 3 P-values of differences in network architecture between 22q11DS and Healthy Controls.
