# Supplementary material for: Development of Structural Covariance From Childhood to Adolescence: A Longitudinal Study in 22q11.2DS
Source: Front Neurosci. 2018 May 18;12:327. doi: 10.3389/fnins.2018.00327 (PMC5968113; doi:10.3389/fnins.2018.00327)
Supplement: Supplementary Table 1 — Demographic characteristics of sample. [file Table_1.DOCX]

|  | Healthy Controls | 22q11DS | P-Value of Difference |
| --- | --- | --- | --- |
| Gender (M/F) | 51/59 | 58/59 | 0.63 |
| Age | 14.48 **^+^**/**_-_** 5.5 | 15.25 **^+^**/ 5.12 | 0.12 |
| Time Between Visits | 3.65 | 3.8 | 0.35 |
| Handness*  (% of Right Handers) | 78% | 73% | 0.61 |
| Full Scale IQ | 110.6 | 70.2 | <0.001 |
| Scanner Type (1.5T/3T_Trio/3T_Primsa) | 78/111/22 | 65/138/20 | 0.14 |
| *Handness is defined using the Edimburgh Laterality Quotient  Between group differences were tested with two samples T-test for the continuous variables and Chi-square test for discrete variables. | | | |
