# Supplementary material for: Development of Structural Covariance From Childhood to Adolescence: A Longitudinal Study in 22q11.2DS
Source: Front Neurosci. 2018 May 18;12:327. doi: 10.3389/fnins.2018.00327 (PMC5968113; doi:10.3389/fnins.2018.00327)
Supplement: Supplementary Table 2 — Prevalence of main psychiatric and neurological diagnoses in 22q11DS sample. [file Table_2.DOCX]

| **Psychiatric and Neurological Diagnoses** | **Prevalence** |
| --- | --- |
| Any Anxiety Disorder | 66 Subjects/ 102 Visits |
| ADHD | 41 Subjects/ 54 Visits |
| Any Mood Disorder | 31 Subjects/ 42 Visits |
| Any Psychotic Disorder | 12 Subjects / 18 Visits |
| Other Psychiatric Diagnoses | 33 Subjects / 39 Visits |
| Lifetime Epilepsy | 7 Subjects |
| Lifetime Isolated Seizures | 8 Subjects |
| The “Other Psychiatric Disorders” category includes oppositional disorders as well as enuresis and encopresis.  ADHD, attention-deficit/hyperactivity disorder; 22q11DS, 22q11.2 deletion syndrome. | |

**Table Legend:**

The presence of DSM-IV psychiatric disorders was assessed by means of the Diagnostic Interview for Children and Adolescents-Revised [1] and the psychosis supplement from the Kiddie Schedule for Affective Disorders and Schizophrenia Present and Lifetime Version [2] for individuals below 18 years of age. For adult participants, we used the Structured Clinical Interview for DSM-IV Axis I Disorders [3].
