# Supplementary figures and images for: Development of Structural Covariance From Childhood to Adolescence: A Longitudinal Study in 22q11.2DS
Source: Front Neurosci. 2018 May 18;12:327. doi: 10.3389/fnins.2018.00327 (PMC5968113; doi:10.3389/fnins.2018.00327)

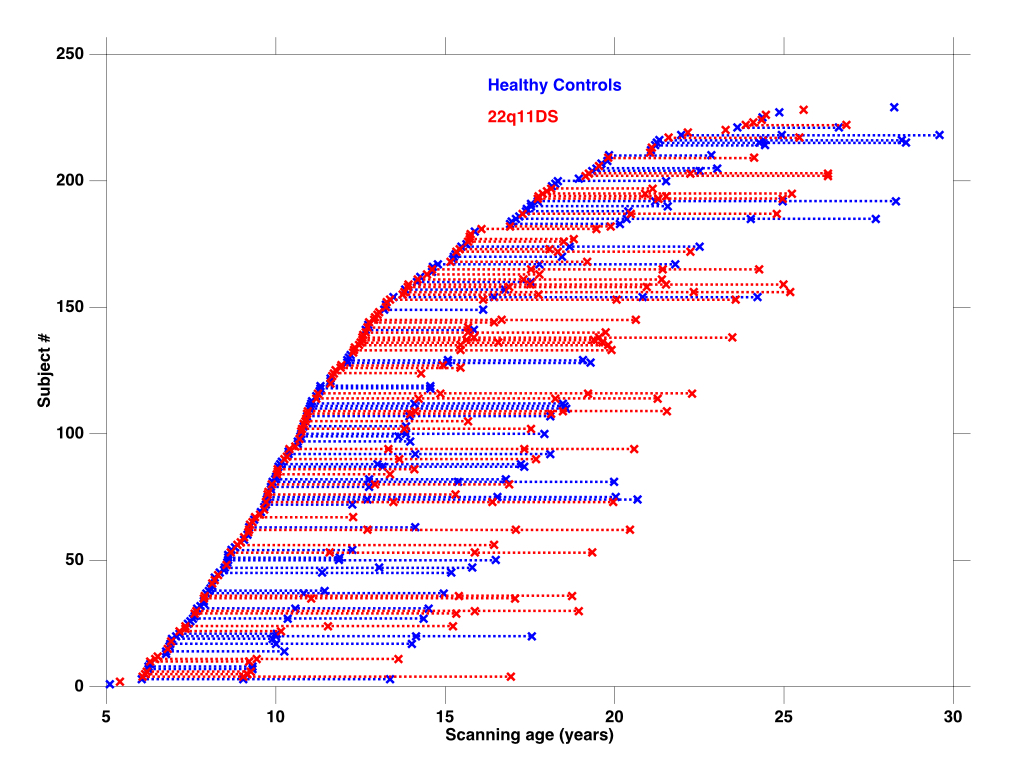

Supplement: Supplementary Figure 1 — Age distribution of sample. [file Image_1.JPEG]

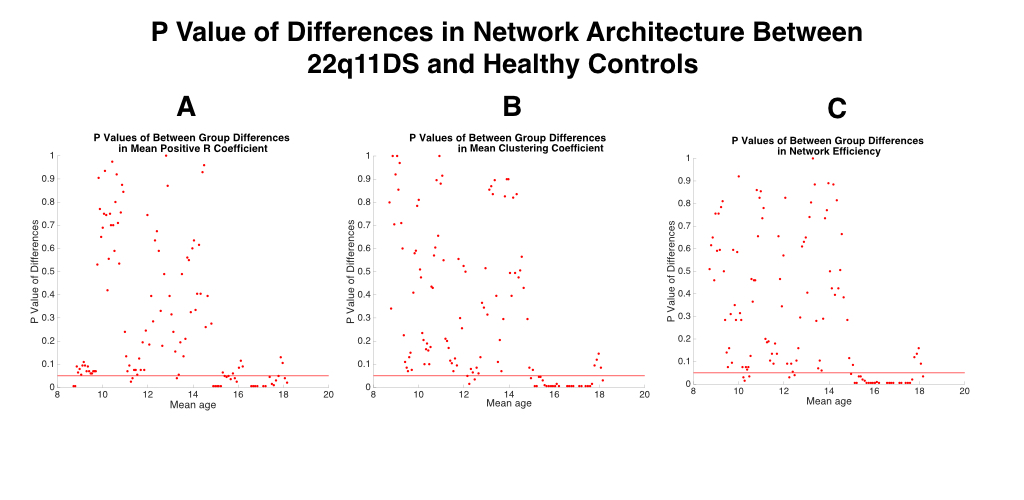

Supplement: Supplementary Figure 3 — P-values of differences in network architecture between 22q11DS and Healthy Controls. [file Image_3.JPEG]
