# Supplementary material for: Development of Structural Covariance From Childhood to Adolescence: A Longitudinal Study in 22q11.2DS
Source: Front Neurosci. 2018 May 18;12:327. doi: 10.3389/fnins.2018.00327 (PMC5968113; doi:10.3389/fnins.2018.00327)
Supplement: Supplementary Figure 2 — Developmental trajectories of local connectivity strength (two cluster solution). [file Image_2.PDF]

Developmental Trajectories of Local Connectivity Strength (2 Cluster Solution)

Healthy Controls

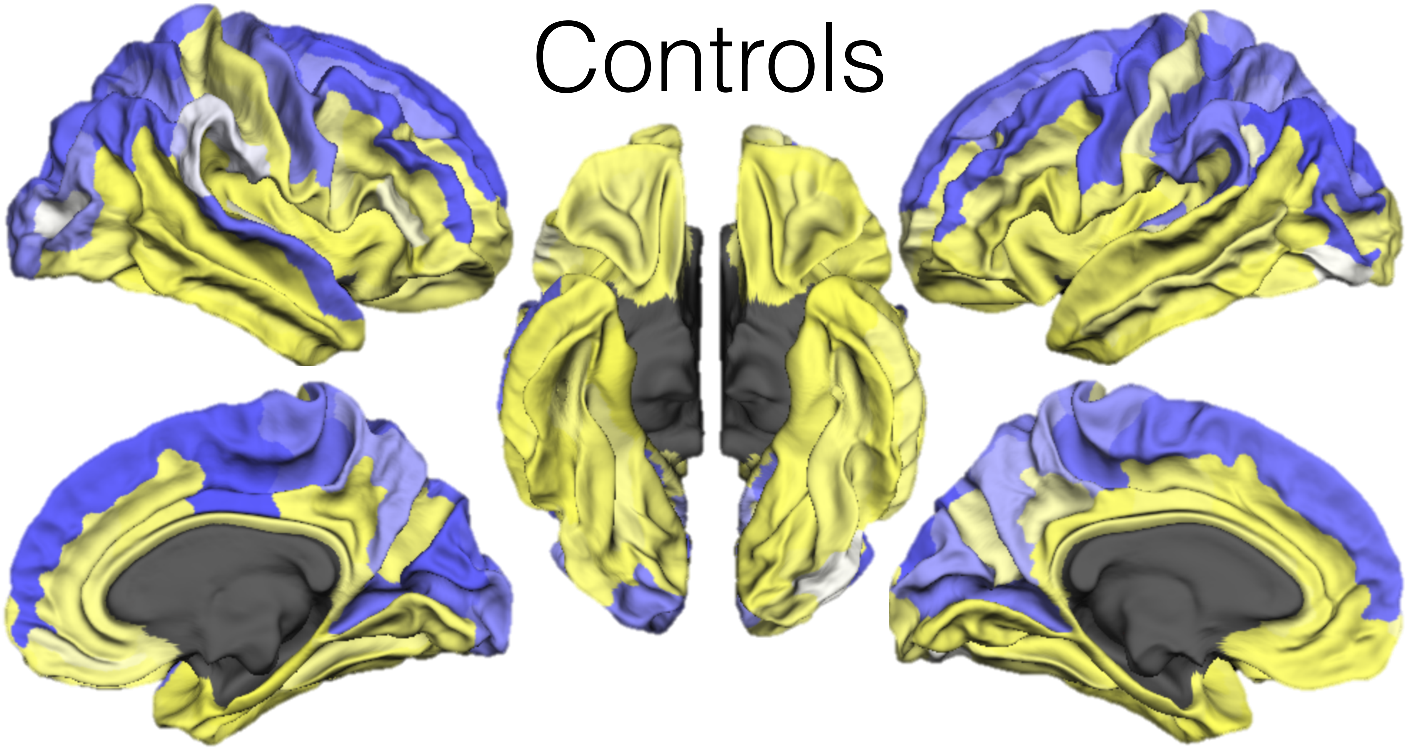

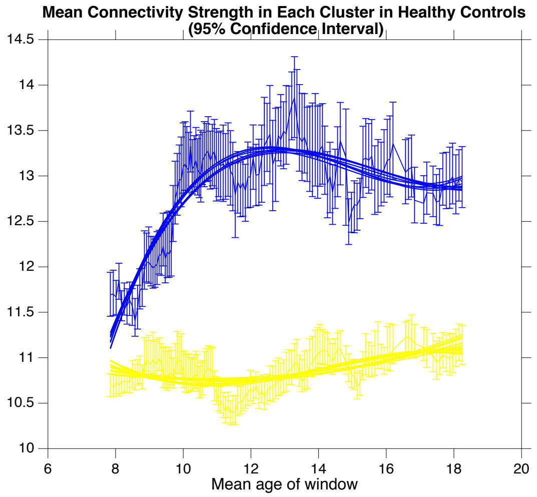

Y=[-12.3 / -7.4] + [4.1 / 5.3] X +
[-0.3/ -0.2] X^2 + [0.005/0.008] X^3
CI of Positive Peak= [12.5-13.0]
CI of Negative Peak= [17.0-18.4]

Y=[15.0 / 16.5] + [-1.3 / -1.1] X +
[-0.07/ -0.1] X^2 + [0.002/0.001] X^3
CI of Negative Peak= [10.5 / 12.9]
CI of Negative Peak= [17.1 / 18.6]
(47% of Bootstrapped Samples)

Y=[11.5 / 12.0] + [- 0.2 / - 0.1] X +
[0.006 / 0.01] X^2
CI of Peak= [10.5 / 11.4]
(53% of Bootstrapped Samples)

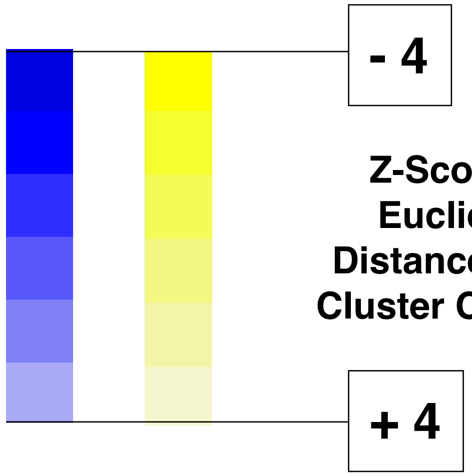

22q11DS

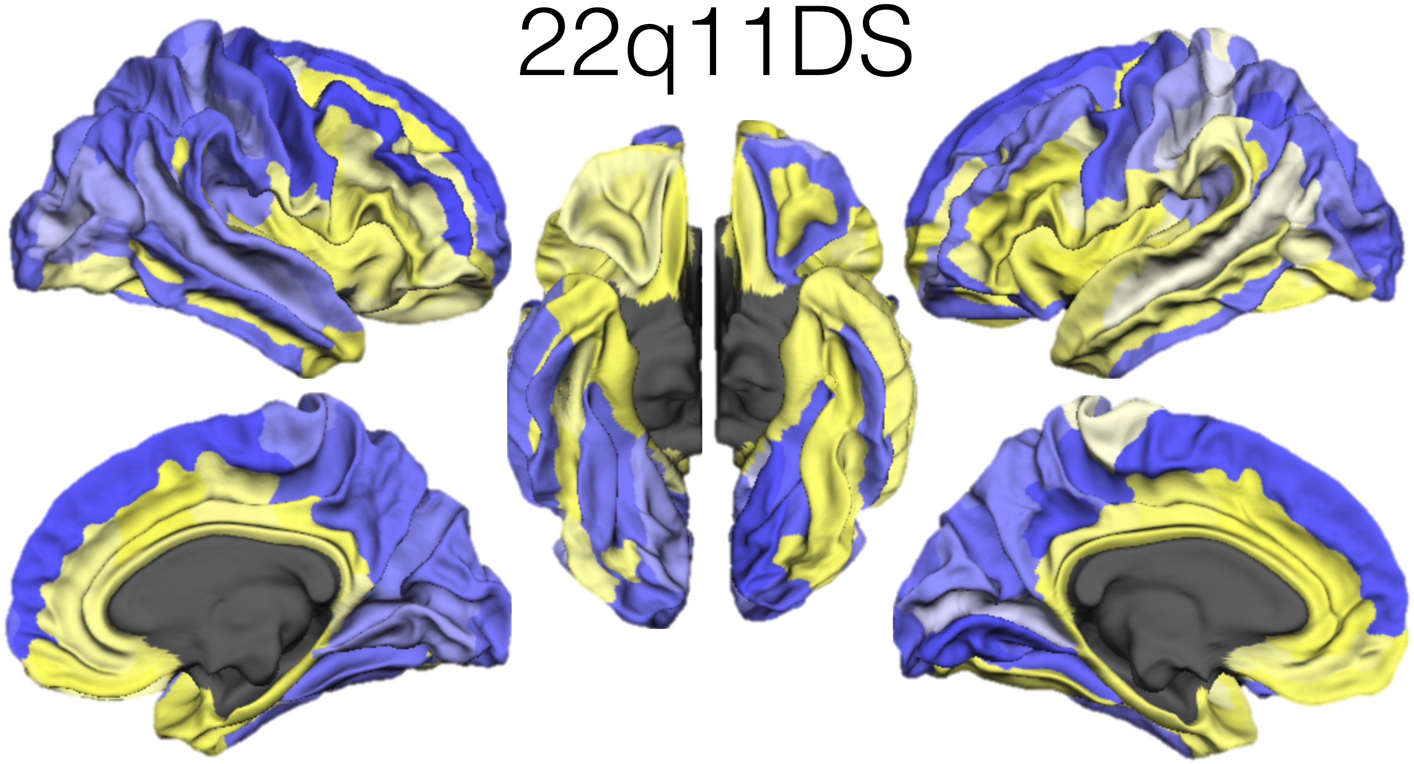

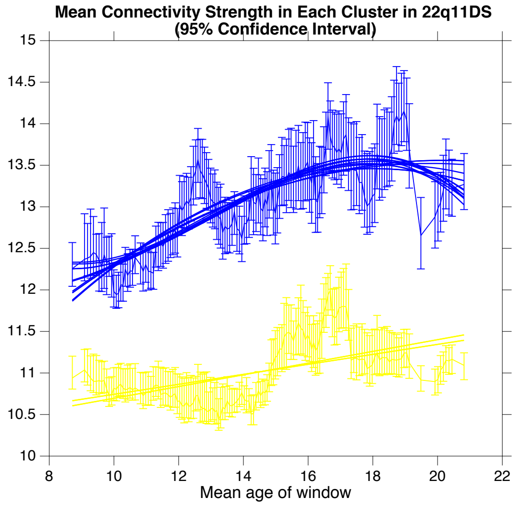

Y=[14.5 / 18.3] + [-1.6 / -0.85] X -
[0.08/ -0.1] X^2 + [-0.003/-0.002] X^3
CI of Negative Peak= [6.8 / 8.8]
CI of Positive Peak= [17.6/ 18.1]
(67% of Bootstrapped Samples)

Y=[7.4 / 8.6] + [0.49 / - 0.1] X +
[-0.018 / -0.012] X^2
CI of Peak= [18.0 / 20.1]
(33% of Bootstrapped Samples)

Y=[9.9 / 10.1] + [0.05 / 0.06] X
